# Frequency Comb-Based Ground-Penetrating Bioradar: System Implementation and Signal Processing

**DOI:** 10.3390/s23031335

**Published:** 2023-01-25

**Authors:** Di Shi, Gunnar Gidion, Taimur Aftab, Leonhard M. Reindl, Stefan J. Rupitsch

**Affiliations:** Laboratory for Electrical Instrumentation and Embedded Systems, Department of Microsystems Engineering—IMTEK, University of Freiburg, Georges-Köhler-Allee 106, 79110 Freiburg, Germany

**Keywords:** respiration detection, bioradar, software-defined radio, ground-penetrating, frequency comb, signal processing, range gating, search and rescue

## Abstract

Radars can be used as sensors to detect the breathing of victims trapped under layers of building materials in catastrophes like earthquakes or gas explosions. In this contribution, we present the implementation of a novel frequency comb continuous wave (FCCW) bioradar module using a commercial software-defined radio (SDR). The FCCW radar transmits multiple equally spaced frequency components simultaneously. The data acquisition of the received combs is frequency domain-based. Hence, it does not require synchronization between the transmit and receive channels, as time domain-based broadband radars, such as ultra wideband (UWB) pulse radar and frequency-modulated CW (FMCW) radar, do. Since a frequency comb has an instantaneous wide bandwidth, the effective scan rate is much higher than that of a step frequency CW (SFCW) radar. This FCCW radar is particularly suitable for small motion detection. Using inverse fast Fourier transform (IFFT), we can decompose the received frequency comb into different ranges and remove ghost signals and interference of further range intervals. The frequency comb we use in this report has a bandwidth of only 60 MHz, resulting in a range resolution of up to 2.5 m, much larger than respiration-induced chest wall motions. However, we demonstrate that in the centimeter range, motions can be detected and evaluated by processing the received comb signals. We want to integrate the bioradar into an unmanned aircraft system for fast and safe search and rescue operations. As a trade-off between ground penetrability and the size and weight of the antenna and the radar module, we use 1.3 GHz as the center frequency. Field measurements show that the proposed FCCW bioradar can detect an alive person through different nonmetallic building materials.

## 1. Introduction

A frequency comb has a series of evenly distributed frequency components and is well-known as a technique for precision spectroscopy [1,2]. In this contribution, we propose using a frequency comb continuous wave (FCCW) as the transmit signal of a so-called bioradar for detecting the breathing of trapped survivors. This work is part of the German civil security research project SORTIE, which aims to develop a lightweight multi-sensor system on board an unmanned aerial vehicle to quickly search and rescue trapped persons in urban disasters like earthquakes.Everyone who is still alive needs to breathe. Respiration causes a periodic expansion and contraction of the thoracic cavity. Optical monitoring technologies like Lidar [3] require the target to be in the line of sight. In contrast, radar can penetrate through materials and detect a covered person. From a radar’s perspective, the radar cross-section (RCS) of the chest wall changes periodically, which results in periodic changes in the radio frequency (RF) power received by the radar.

The most basic bioradar is the continuous wave (CW) radar, which transmits and receives continuous waves of a single frequency. One primary research focus of quadrature CW radar is to reduce the in-phase and quadrature-phase (IQ) imbalance and eliminate the direct current (DC) offset for accurate phase demodulation. Studies on heterodyne demodulation architectures [4], six-port interferometers [5], frequency group radars [6], and nonlinear channel combining algorithms [7] have shown promising results for solving this problem. After center and imbalance correction, the Doppler phase shift of the CW signal can be accurately demodulated to determine the small, time-varying displacement of the chest wall due to breathing. Studies report the use of CW radar to non-contact monitor the vital signs of children [8] and hospitalized patients [9,10], identify abnormal breathing patterns [11], diagnose elevated blood pressure [12], and even authenticate users based on cardiac motion information [13]. Researchers in [14] provide a comprehensive review of the progress made in CW-based bioradars over the past decade. The main drawback of a CW radar is that it cannot provide any range information, so it can neither range the victim nor eliminate ghost signals from another distance. For the aforementioned applications, distance information is not essential.

Wideband bioradars are more complicated to implement but can provide distance information. According to the transmitted waveform, they can be classified as ultra wideband (UWB) impulse radar, frequency-modulated CW radar (FMCW), step frequency CW radar (SFCW), and so on. In [15], the latest applications of bioradar technology are surveyed, especially those using wideband bioradars. A UWB impulse radar transmits short pulses with ultra wide bandwidth and high energy. UWB impulse radar has strong penetrating ability and is widely used in ground-penetrating radar (GPR) applications. Using the time-of-flight (TOF) principle, a UWB impulse radar can determine a target’s range by measuring its echo’s time delay. By comparing the delay over slow time, it can accurately determine the Doppler shift of the target. In many investigations on bioradar, UWB impulse radar is utilized [16,17,18,19,20]. Due to its ultra wide bandwidth, the range resolution can be on the order of centimeters [15]. However, receiving the short UWB echo and preserving the pulse waveform require very high-speed analog-to-digital converters (ADCs) [21,22], which are expensive and consume higher computational power. Additionally, UWB impulse radar requires the antenna to have high gain in a continuous wide band. A typical FMCW radar emits a signal with monotonically increasing or decreasing frequency, and its instantaneous bandwidth is narrow. By mixing the transmitted and received signals, a frequency or phase difference can be obtained [23,24]. Both UWB impulse radar and FMCW radar are TOF-based and require strict synchronization between the transmitter and receiver for correct ranging.

An SFCW radar sweeps the carrier frequency step-wise and can cover a wide frequency band, and its signal is instantaneously narrowband. Compared with UWB impulse radars, an SFCW radar uses low-speed ADCs [15,21], has more flexibility in antenna choice, and is easier to calibrate [25]. A vector network analyzer (VNA) is a standard instrument that exploits radio frequency sweeps to generate and receive signals. VNAs are often utilized for proof-of-concept bioradar studies using SFCW, especially under laboratory conditions [25,26,27]. The frequency sweep process is also known as frequency hopping. The sweep repetition rate of the carrier frequency is the actual sampling rate in moving target detection. This frequency sweep process is a double-edged sword. On the one hand, SFCW radar takes more time to transmit and sample the signal of one frequency, making the echo measurement more accurate and achieving higher SNR in respiration detection [22,25]. On the other hand, this sweeping process is time-consuming. For each frequency step, the phase-locked loop (PLL) needs time to stabilize [28].

Another technique that uses equally spaced frequencies is orthogonal frequency division multiplexing (OFDM). OFDM is first and foremost a digital transmission method that uses multiple mutually orthogonal carrier frequencies so that signals with overlapping spectra can be transmitted without crosstalk between the subchannels [29]. One drawback of OFDM in digital communication is its sensitivity to time and frequency synchronization offsets between the transmitter and receiver. However, this sensitivity can be used in radar applications for more accurate target detection measurements [30].

Our frequency comb is automatically an OFDM signal, and it is a continuous wave in the time domain. Unlike OFDM signals, the tones in a frequency comb do not have overlapping spectra. We will show that when two mathematical requirements are met, a digital waveform can have a clean frequency comb spectrum of only ones and zeros. In this contribution, we report the implementation of an FCCW bioradar module utilizing a small-size commercial software-defined radio (SDR). To our best knowledge, this is the first work reporting a purely software-defined FCCW signal for ground-penetrating bioradar applications. This FCCW radar transmits and receives simultaneously without frame synchronization, requiring only a phase lock between the receiver’s local oscillator (LO) and the transmitter’s LO, enabling a much higher scan rate.

We present the signal processing of the received signal by taking a laboratory measurement as an example. The received frames are treated with several transforms and are decomposed into different distance intervals. The respiratory frequency of the trapped person is determined using the fast Fourier transform (FFT) and the continuous wavelet transform (CWT), respectively. Based on empirical results, we have defined the respiratory signal prominence ratio and the normalized difference of estimated respiratory rates to evaluate the detection quality of respiration. We use these two metrics to determine whether life is detected in a particular measurement.

The article is organized as follows. In Section 2, the basic bioradar measuring principle, the generation of the frequency comb, and the ground penetrating bioradar are introduced. Section 3 describes the SDR bioradar system’s construction and defines the radar specifications. In Section 4, taking one measurement as an example, the signal processing procedure is introduced step by step. Section 5 presents an experiment with two test persons. In Section 6, the field experiments in four realistic disaster scenarios are conducted and analyzed. Section 7 summarizes the main contributions of this work and gives an outlook on future work.

## 2. Basics

### 2.1. Bioradar Measuring Principle

**Figure 1:**
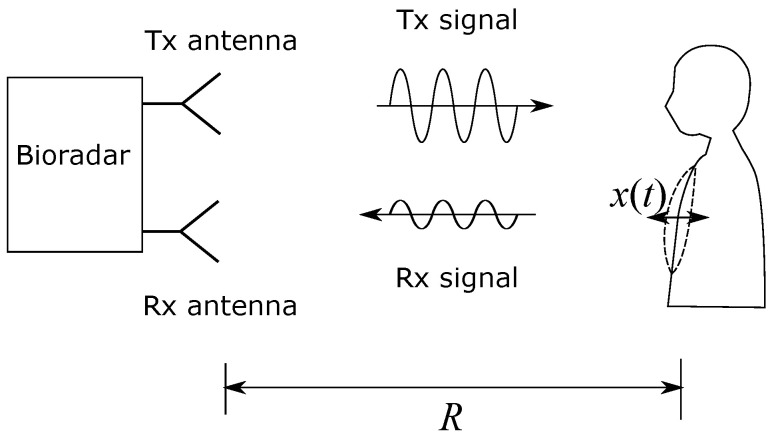
Illustration of the bioradar measuring principle.

Humans breathe at rest 6 to 45 times per minute [31,32,33,34], which corresponds 0.1 to 0.75 Hz. Infants breathe fastest, and with increasing age, children breathe slower until adulthood [32]. On average, our respiratory rate $f_{\mathrm{resp}}$ during sleep is slower than during wakefulness [33,34]. The measuring principle of the bioradar is based on the Doppler effect [8]. As illustrated in Figure 1, the bioradar transmits and receives electromagnetic waves. A person is at a distance *R* from the transmitter and receiver antennas.

When a person breathes at the respiratory rate $f_{\mathrm{resp}}$, the distance *R* between the radar system and his chest will change periodically. We define the change in distance as x(t). Simplified, one can represent x(t) as a sine function with $f_{\mathrm{resp}}$ [8]

(1) $$x\left(t\right)=x_{\max}\sin\left(2\pi f_{\mathrm{resp}}t\right).$$

The change in the distance leads to a phase change ϕ in the reflected waves and results in a shift in frequency, which is known as the Doppler frequency $f_{D}$ [35]

(2) $$f_{D}=\frac{d\phi (t)}{2\pi dt}=\frac{2}{\lambda }v\left(t\right).$$

where v(t) is the velocity of the chest movement

(3) $$v\left(t\right)=\frac{dx(t)}{dt}=2\pi f_{\mathrm{resp}}x_{\max}\cos\left(2\pi f_{\mathrm{resp}}t\right).$$

Therefore, the Doppler frequency $f_{D}$ changes periodically with the respiratory frequency $f_{\mathrm{resp}}$. During a one-minute measurement, $f_{\mathrm{resp}}$ remains roughly constant, whereas the value and sign of $f_{D}$ change periodically. In addition to the change in distance, the radar cross-section of the chest also changes. As a result, both the phase ϕ of the reflected radar waves and the amplitude *A* change with $f_{\mathrm{resp}}$.

### 2.2. Frequency Comb CW Radar

A single carrier frequency $f_{\mathrm{tx}}$ is sufficient to measure the breathing rate. Additional information about the distance from the radar to the Doppler signal source helps to distinguish a potentially buried person from other sources, such as a first responder or tree branches moved by wind. To obtain a distance resolution ΔR, the measurement signal must feature a certain bandwidth BW.

The bioradar of the SORTIE project uses a frequency comb, which is generated by a continuous waveform with *M* evenly distributed discrete frequency components $f_{\mathrm{tx}}\left[m\right]$ in the baseband,

(4) $$f_{\mathrm{tx}}\left[m\right]=f_{0}+\left(m-1\right)\Delta f_{\mathrm{tx}},\;\mathrm{for}\;m=1,2,\ldots M,$$

with the start frequency $f_{0}=\left(-M/2+0.25\right)\Delta f_{\mathrm{tx}}$ and the frequency step between two neighboring components $\Delta f_{\mathrm{tx}}=BW/\left(M-1\right)$. The continuous waveform s[l] is the superposition of all $f_{\mathrm{tx}}\left[m\right]$ waves

(5) $$s\left[l\right]=\sum_{m=1}^{M}\cos\left(2\pi f_{\mathrm{tx}}\left[m\right]\frac{l}{f_{s}}\right)+j\sin\left(2\pi f_{\mathrm{tx}}\left[m\right]\frac{l}{f_{s}}\right),$$

where *l* is the index of the samples in the time domain. This frequency comb is defined in the baseband and has negative frequencies. By transmitting, the comb will be mixed with a carrier frequency and sent in a high-frequency band. The offset of $0.25\Delta f_{\mathrm{tx}}$ ensures that the frequency comb has no component at 0 Hz and, therefore, does not crosstalk with the carrier frequency. The slight asymmetry around 0 Hz also makes s[l] have an imaginary part.

In theory, the Fourier transform of a Dirac comb signal is also a Dirac comb. In the case of digital signals, however, due to discretization, the tones may have different amplitudes, and noise may appear between the comb tones. If the parameters of the frequency comb fulfill the following two requirements, we can maximally use the sampling frequency $f_{s}$ and avoid errors caused by discretization in both time and frequency domains. The first requirement asks the comb to divide the sampling frequency $f_{s}$ evenly.

- **Requirement 1**:
  (6) $$f_{s}=M\;\cdot \;\Delta f_{\mathrm{tx}}.$$

This maximally uses the $f_{s}$ and ensures that the sampling theorem is not violated, since the bandwidth of the comb $BW=f_{s}\left(M-1\right)/M<f_{s}$. Transforming the analog frequency *f* to the digital domain, the normalized digital angular frequency $\omega =2\pi f/f_{s}$, and $f_{s}$ corresponds to ω=2π. In a complex plane, the comb equally divides the unit circle into *M* parts. If we write s[l] of Equation (5) in complex exponential form, we obtain

(7) $$s\left[l\right]=\sum_{m=1}^{M}e^{j\omega l\left(-\frac{M}{2}+0.25+\left(m-1\right)\right)\frac{2\pi }{M}}=e^{j\omega l(-\pi -\frac{1.5\pi }{M})}\sum_{m=1}^{M}e^{j\omega l\frac{2\pi }{M}m}.$$

**Figure 2:**
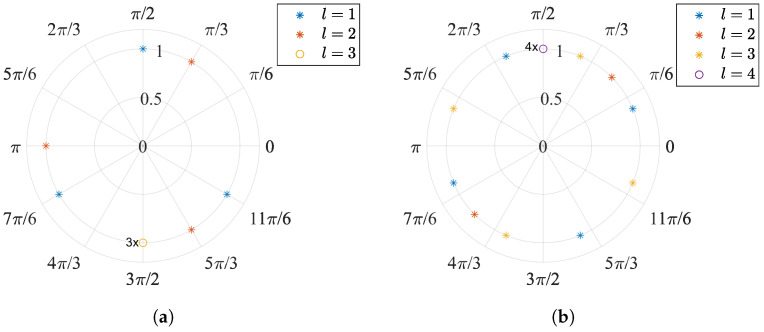
Polar plot of two s[l] examples: (**a**) M=3, odd. The comb has three tones. For l=1 and 2, these tones equally distribute on the unit circle, and their complex sum is zero. For l=3, the three tones overlap at −j. (**b**) M=4, even. The comb has four tones. For l=4, the four tones overlap at j.

For the sample *l*, the summation term has two possibilities. As shown in Figure 2, when l≠kM, with $k\in Z^{+}$, the *M* frequency components evenly distribute *l* unit circles and compensate each other, making their complex summation zero. By every *M*th sample, i.e., l=kM, all *M* frequency components meet at one point on the unit circle and build a peak with a magnitude of *M*.

Regardless of the frequency number *M*, there are two possibilities for the summation term of s[l],

(8) $$\sum_{m=1}^{M}e^{j\omega l\frac{2\pi }{M}m}=\{\begin{array}{cc} 0, & \;\mathrm{for}\;l\neq kM;\\ M, & \;\mathrm{for}\;l=kM; \end{array}\;\;\;\mathrm{with}\;k\in Z^{+}.$$

The offset of $0.25\Delta f_{\mathrm{tx}}=0.25\frac{2\pi }{M}=\frac{0.5\pi }{M}$ makes the peaks in s[l] appear only on the real or the imaginary axis. For non-zero summation terms, i.e., l=kM,

(9) $$\begin{array}{c} e^{j\omega kM(-\pi -\frac{1.5\pi }{M})}=\{\begin{array}{cccc} e^{j\omega k0.5\pi } & = & j^{k}, & \;\mathrm{for}\;M\;\mathrm{is}\;\mathrm{even};\\ e^{-j\omega k0.5\pi } & = & {\left(-j\right)}^{k}, & \;\mathrm{for}\;M\;\mathrm{is}\;\mathrm{odd}. \end{array} \end{array}$$

Substituting Equations (8) and (9) in Equation (7), we get

(10) $$s\left[l\right]=\{\begin{array}{cccc} 0, & \;\mathrm{for}\; & l\neq kM; & \\ j^{k}M, & \;\mathrm{for}\; & l=kM, & M\;\mathrm{is}\;\mathrm{even};\\ {\left(-j\right)}^{k}M, & \;\mathrm{for}\; & l=kM, & M\;\mathrm{is}\;\mathrm{odd}. \end{array}$$

Accordingly, s[l] has five possible values: [0,±M,±jM]. For an s[l] with kM samples, k(M−1) samples are zeros. The *k* non-zero samples are either purely real or imaginary, with magnitude *M*. The second requirement makes the wave in the analog domain a continuous wave:

- **Requirement 2**:
  (11) L=k·4M,withk∈N.

**Figure 3:**
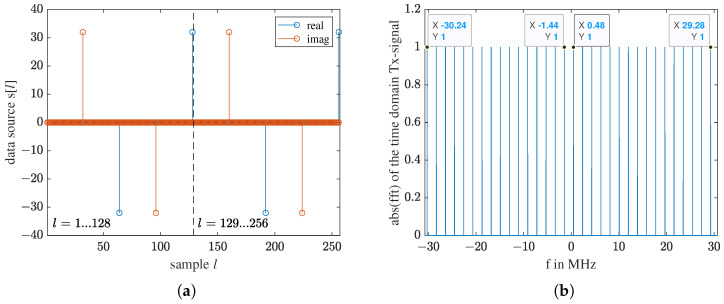
For transmitting: (**a**) The pre-defined waveform in sample time domain. Samples l=1…128 built one complete wave. (**b**) The spectrum of the waveform s[l] with a length L=4096.

*L* is the length of s[l]. With 4M samples, s[l] makes one full circle, forming one complete wave. If *L* is an integer multiple of 4M, the transmitted wave will have no jump discontinuity between one *L* and the next *L*, making it continuous. So, in contrast to an FMCW chirp modulation, using this FCCW modulation, the receiver can start sampling anytime without synchronization with the transmitter. Figure 3a shows the waveform s[l] for M=32. L=4·32=128 samples build a complete wave. To achieve a sharp comb in the frequency domain, we increase *L* to 4096. Figure 3b shows the resulting frequency comb. Table 1 lists the specifications of the frequency comb used in this work.

### 2.3. Ground-Penetrating Bioradar

For survivor detection applications, bioradars must operate at low frequencies to penetrate layers of collapsed building materials well [36,37]. On the other hand, low frequencies require physically large radar antennas, which makes it challenging to integrate the system into an unmanned aircraft system (UAS). As a trade-off, we use 1.3 GHz as the center frequency. For this research work, BNetzA (Federal Network Agency, Bonn, Germany) granted the authors access to the radio spectrum from 1.26 GHz to 1.34 GHz.

**Figure 4:**
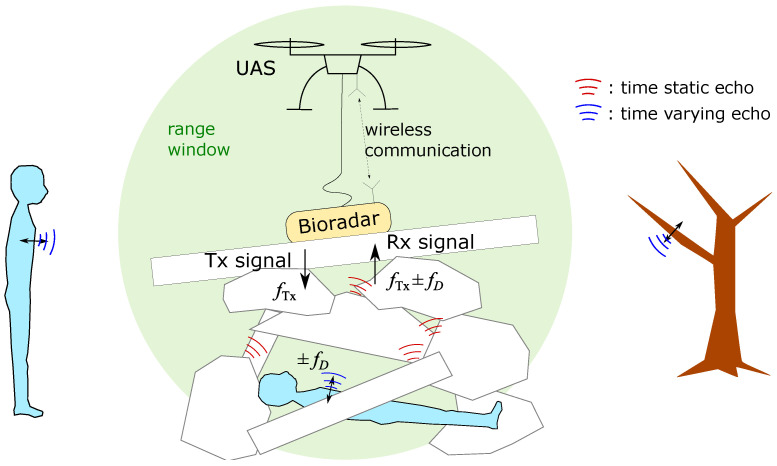
Illustration of a bioradar operation scenario. A person is trapped underneath rubble piles. A bioradar is on top of the rubble pile. All objects in the environment reflect the transmitted radar signal. A non-trapped person and a vibrating tree are out of the range window.

Figure 4 illustrates the employment scenario of a bioradar at a disaster site. There is a person trapped underneath layers of rubble piles. If the transmit (Tx) and receive (Rx) antennas of the bioradar are in direct contact with the rubble surface, air–ground reflections can be minimized and better ground penetration can be achieved. The distribution of the building materials is unknown and complex, and the materials are dispersive media. Regardless of the waveform, penetrating through these materials will introduce dispersion noise into the wave. Additionally, there might be some vibration sources in the ambiance causing ghost signals, for example, a rescuer or some vibrating tree branches.

However, if these interferers are in another distance interval, we can use a range window to separate them from the breathing signal of the trapped person. The range resolution ΔR depends on the radar bandwidth BW in use,

(12) $$\Delta R=\frac{c}{2\;\cdot \;BW},$$

where *c* is the propagation velocity of electromagnetic (EM) waves. Since the composition of the material and cavity under the surface of the rubble is unknown, it is impossible to give an accurate actual ΔR. What can be determined is the maximum possible range interval length, which is the range resolution in air, $\Delta R_{\max}=c_{0}/\left(2\;\cdot \;BW\right)$, where $c_{0}$ is the EM wave velocity in the air. Combined with the number of transmitting frequencies *M*, we can calculate the maximum unambiguous range $R_{\max}$ of the radar,

(13) $$R_{\max}=\frac{M}{2}\;\cdot \;\Delta R.$$

$R_{\max}$ describes the distance limit in the space that does not cause aliasing. In Section 2.2, we chose M=32 because it gives a maximum possible range resolution of $\Delta R_{\max}=\left(3\;\cdot \;10^{8}\;m/s\right)/\left(2\;\cdot \;59.52\;\mathrm{MHz}\right)\approx 2.52\;m$ and a maximum unambiguous range $R_{\max}=32/2\times 2.52\;m=40.32\;m$. The speed of the RF waves in dielectric materials mainly depends on the relative permittivity of the medium. Suppose the effective relative dielectric constant of the ground material is $\epsilon_{r}=4$. In that case, the speed of the RF wave in the medium will be reduced by a factor of $\sqrt{4}$, resulting in a maximum unambiguous range of $R_{\epsilon_{r}4,\max}=R_{\epsilon_{r}1,\max}/\sqrt{4}=20\;m$. For ground-penetrating bioradar application, most interference beyond 20 m is negligible.

## 3. System Description: SDR-Based Bioradar

In the previous FOUNT^2^ project, the bioradar system was integrated into a UAS. The antennas were built into the feet of the landing gear to ensure direct contact with the ground after landing [38]. However, a safe landing spot for UAS cannot be found on all debris fields. To overcome this problem, in the SORTIE project, we propose to design a standalone ground-penetrating radar (GPR) that can be unloaded on the rubble by the UAS with a rope without landing. The UAS communicates with the bioradar wirelessly.

**Figure 5:**
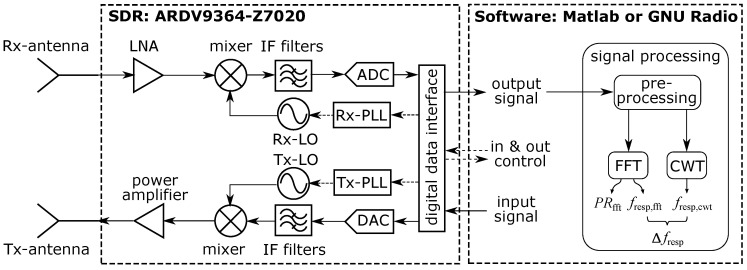
Block diagram of the software-defined radio bioradar. The SDR block is modified from [40]. Next Section shows a detailed version of the signal processing process.

A software-defined radio (SDR) is a radio in which some or all physical layer functions are software-defined [39]. Without deep radio hardware design knowledge, academic researchers and industrial engineers can easily change the hardware parameters of an SDR through the software. As shown in the simplified block diagram in Figure 5, an SDR-based bioradar has three main parts. In the front, the Tx antenna acts as an actuator, and the Rx antenna as a sensor. In the middle, the SDR hardware connects the analog and digital worlds. At the other end, the software defines the SDR’s physical layer settings and collects data.

We use the wideband SDR ADRV9364-Z7020 from Analog Devices (Wilmington, MA, USA). The RF transceiver of this SDR uses a direct conversion architecture with only one intermediate frequency (IF) mixing stage [41]. As a result, it can be implemented very compactly in terms of size and weight [39]. Additionally, the receiver’s local oscillator (LO) and the transmitter’s LO are phase-locked by the integrated phase-locked loops (PLL). The important system parameters for the SORTIE bioradar are summarized in Table 2. The LO frequencies of the transmitter and the receiver are set to different values to prevent carrier leakage.

In a bulletin issued by the US Federal Communications Commission (FCC), the radio frequency field exposure limit for the general population is defined as 0.08 W/kg as averaged for the whole body, and the spatial peak shall not exceed 1.6 W/kg [42]. The maximum transmitter output power of the transceiver ADRV9364 (Wilmington, MA, USA) is 8 dBm [40], corresponding to 6.31 mW. Even with a high gain antenna and the person positioned directly in front of the antenna, the maximum transmitted power of the SDR is below the exposure limit associated with potentially adverse biological effects.

## 4. Signal Processing

**Figure 6:**
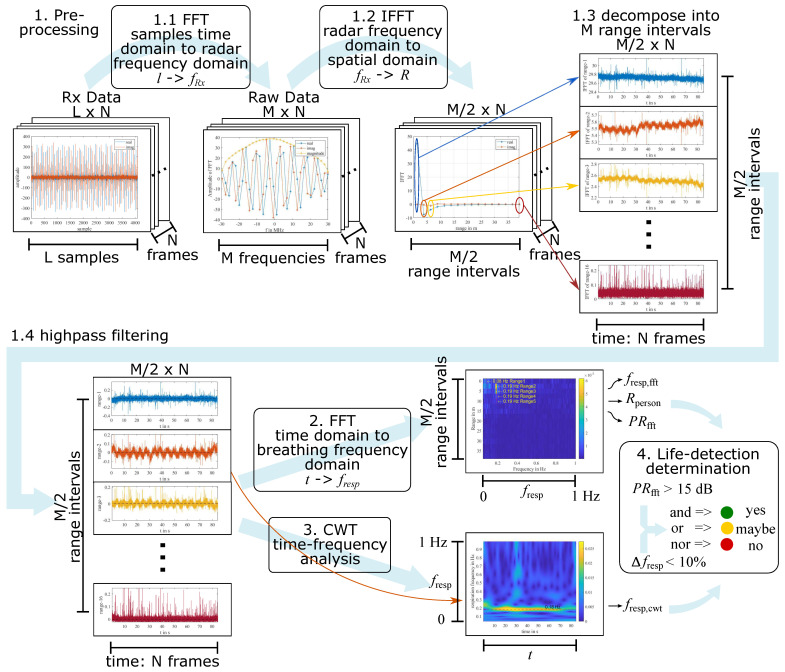
Signal processing procedure. Step 1, pre-processing, includes four sub-steps: *N* times FFT, *N* times IFFT, decompose the signal into *M* range intervals and keep the first M/2 sub-signals, and highpass filter them. In step 2, another FFT is applied to these sub-signals, respectively. The result of the second step is a plot and three values. Step 3 calculates the time-frequency distribution of the sub-signal from $R_{person}$ and results in a diagram and two values. With the results from steps 2 and 3, the decision about whether life is detected can be determined in Step 4.

The aim of signal processing is to find and evaluate the breathing signal and determine whether an alive person is detected in a measurement. There are four main steps in the proposed signal processing algorithm. The whole procedure is summarized in Figure 6.

**Figure 7:**
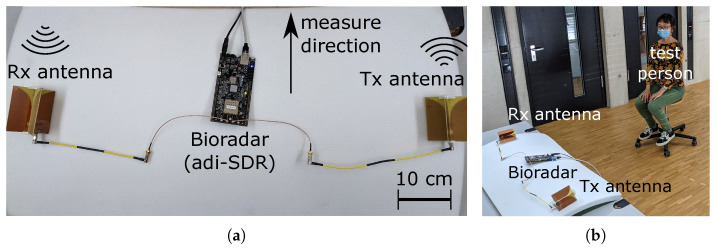
(**a**) Photograph of the Bioradar (adrv9364-z7020) with two Vivaldi antennas. (**b**) A test person sits in front of the test setup.

In the following, we use a laboratory measurement as an example to explain the signal processing process. Figure 7 shows a basic bioradar test setup with an ADRV9364-Z7020 SDR (Wilmington, MA, USA) and two Vivaldi antennas (manufactured in our laboratory). The SDR communicates with the host laptop through an Ethernet cable. A test person sits in front of the test setup.

### 4.1. Pre-Processing: Radar Frequency Domain to Spatial Domain

**Figure 8:**
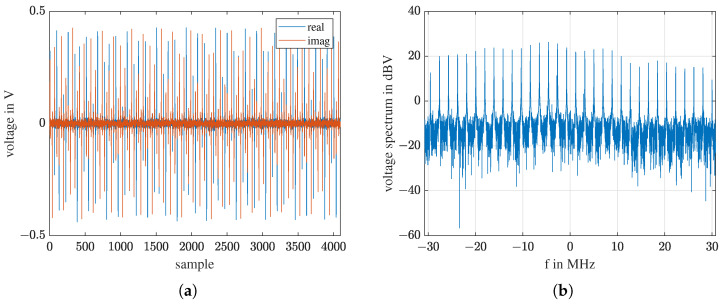
For receiving: (**a**) One frame of the received signal. (**b**) The spectrum of this frame.

In the receive path, the reflected signal is sampled as a series of complex numbers and stored in *N* frames, with *L* samples per frame. The frequency comb is extracted from the broadband sampled signal in the first processing step. To do this, for each frame $s_{\mathrm{Rx}}\left[l\right]$, with l=1…L, the frequency spectrum $S_{\mathrm{Rx}}$ is computed using a fast Fourier transform (FFT),

(14) $$S_{\mathrm{Rx}}\left[k\right]=\frac{1}{L}\sum_{l=1}^{L}s_{\mathrm{Rx}}\left[l\right]e^{-j\frac{2\pi }{L}kl},$$

where *k* is the index of frequency, k=1⋯L. Only the *M* frequencies used in the frequency comb are further processed. Due to a speed mismatch between the host computer and the SDR [39], there is frame loss. One frame of the received signal is shown in Figure 8a, and its spectrum is shown in Figure 8b. The spectrum covers the 61.44 MHz sampling band, meaning no samples are lost in one frame. It is acceptable for our application as long as frame loss occurs periodically. If a measurement has *N* frames, the dimension of the raw data is M×N.

**Figure 9:**
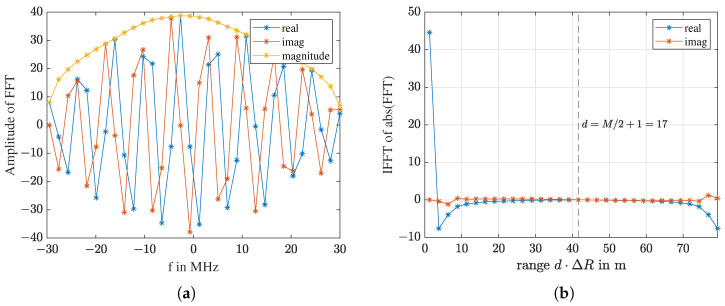
(**a**) The 32 points of raw data of one frame, linear scale of Figure 8b. (**b**) The IFFT of the magnitude in (**a**).

With an inverse Fourier transformation (IFFT), the spectrum can be transformed from the frequency domain to the fast-time domain. For *M* discrete frequencies, one thus obtains *M* distances in the spatial domain

(15) $$x\left[d\right]=\sum_{m=1}^{M}\left|S_{\mathrm{Rx}}\left[m\right]\right|e^{j\frac{2\pi }{M}md},\mathrm{with}\;d=1\ldots M.$$

where *d* is the index of range and $|S_{\mathrm{Rx}}\left[m\right]|$ is the magnitude of $S_{\mathrm{Rx}}\left[k\right]$ at the *m*-th transmitting frequency, as indicated by the spikes in Figure 8b and Figure 9a. With the distance resolution ΔR (Equation (12)), the wave traveling time, the so-called fast-time, can be converted into a spatial distance d·ΔR. Figure 9b shows the complex-valued IFFT of the magnitude spectrum in Figure 9a. The first value of the IFFT is real and has the largest magnitude. The rest of the M−1 values are complex symmetric around d=M/2+1. The first M/2 values contain all the information.

**Figure 10:**
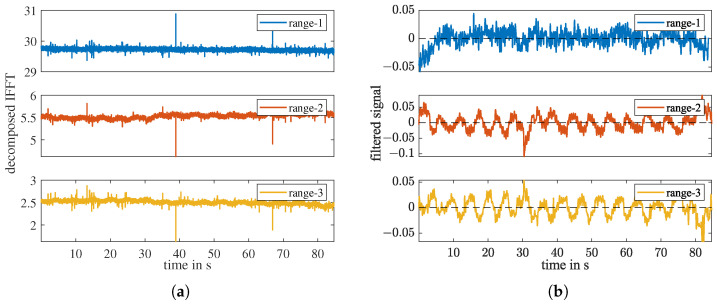
(**a**) The real part of the time domain decomposed signal for the first three ranges of the measurement shown in Figure 7. Range-1 ≈0 to 2.5 m, range-2 ≈2.5 to 5 m, range-3 ≈5 to 7.5 m. (**b**) The corresponding filtered signals.

In the third sub-step, the signal is decomposed into different distances. For each distance range, d·ΔR, the *d*-th point in the IFFT x[d] forms a time domain signal $x_{d}\left[n\right]$ for this distance over all *N* frames. Figure 10a shows the sub-signals of the first three range intervals, which are noisy and have static offsets. In the fourth sub-step, we apply a 4090-order FIR highpass filter with cutoff-frequency $f_{c}=0.05\mathrm{Hz}$ on these sub-signals. As shown in Figure 10b, after the highpass filtering, all the sub-signals are vertically centered around 0, and the sub-signals of range-2 and -3 exhibit apparent periodicity.

### 4.2. FFT: Time Domain to Breathing Frequency Domain

In Step 2, we apply a second FFT on the pre-processed signals, respectively, and convert them from the time domain to the breathing frequency domain. The FFT of the signal of the *d*-th range $x_{d}\left[n\right]$ is

(16) $$X_{d}\left[k\right]=\sum_{n=0}^{NFFT-1}x_{d}\left[n\right]e^{-j\frac{2\pi }{NFFT}kn},$$

with NFFT being the next power of 2 of *N*. $x_{d}\left[N+1\right]$ to $x_{d}\left[NFFT\right]$ are zero-padding. We only analyze the frequency range between 0.08 Hz and 1 Hz, since no one breathes slower than 0.08 Hz or faster than 1 Hz. The frequency with the highest amplitude in this frequency interval gives the respiration rate calculated via FFT,

(17) $$f_{\mathrm{resp},\mathrm{fft}}=f(|X_{\max}\left|\right),\mathrm{for}\;f\in \left[0.08,1\mathrm{Hz}\right].$$

**Figure 11:**
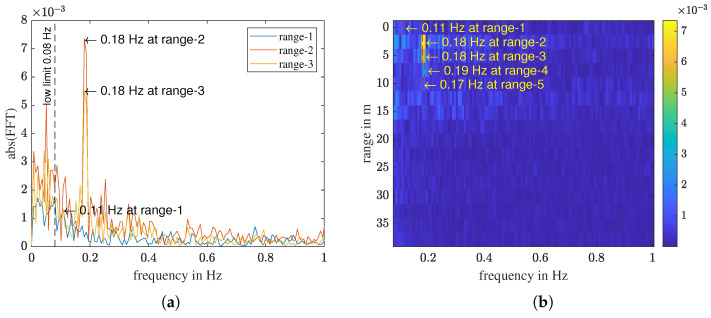
(**a**) FFT of the filtered signals of the first three range bins shown in Figure 10; (**b**) The breathing frequency–range plot.

Figure 11a shows the FFT of the three sub-signals in Figure 10b. If multiple range intervals exhibit a maximum at the same frequency, the person locates in the first range of these intervals. Signals in farther intervals are multipath reflections of the person by objects like the ground and walls in the room. From this plot, we can tell that the person locates in the second range interval because the sub-signals of range-2 and range-3 have the maximum at the same frequency 0.19 Hz. All three sub-signals have some static offset remainders in the frequency range close to 0 Hz. The maximum FFT magnitude of range-1 is in the remainder noise. The FFT of all range intervals can be plotted together and results in a respiratory frequency–range plot as shown Figure 11b. Each horizontal color strip is the FFT of one range interval. The maximum values of the first five range intervals are marked on the graph. Multipath reflections can be seen from range-3 to range-5, with decreasing intensity.

To describe the quality of the breathing signal in the FFT spectrum, we define the prominence ratio $PR_{\mathrm{fft}}$, which is the power of the highest amplitude compared to the average power of other points in the frequency range [0.08, 1 Hz]:

(18) $$PR_{\mathrm{fft}}=\frac{P_{\mathrm{signal}}}{P_{\mathrm{noise}}}=\frac{X_{\max}^{2}}{(\sum_{k=1}^{L}|X\left(k\right){|}^{2}-X_{\max}^{2})/\left(L-1\right)},\mathrm{for}\;f\in \left[0.08,\;1\mathrm{Hz}\right].$$

The AD9364 transceiver is equipped with an automatic gain control system (AGC), which can maintain the receiving signal power at a certain level. However, to use the prominence ratio as a metric of the detection quality, the AGC should be disabled. Here, we fix the receiver gain as 50 dB.

### 4.3. Continuous Wavelet Transform (CWT): Time–Frequency Analysis

**Figure 12:**
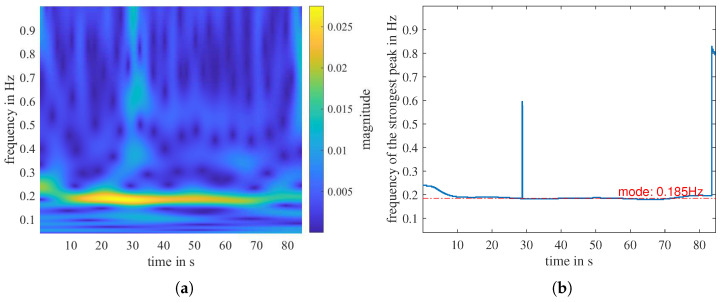
The CWT time-frequency distribution of the range-2 signal in Figure 10b. (**a**) The distribution for frequency f∈ [0, 1 Hz]. (**b**) The 1D representation of (**a**): the strongest peak of each time point. The mode value of the curve is marked in red.

**Figure 13:**
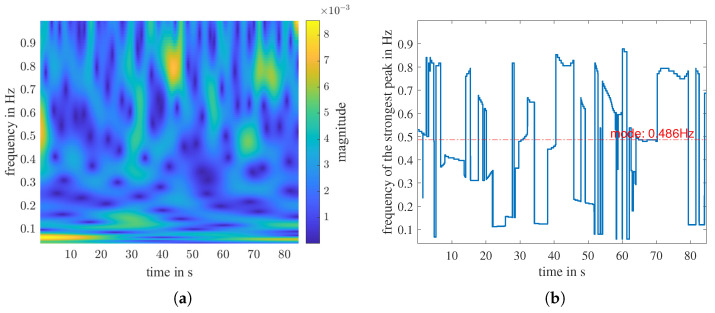
The CWT time–frequency distribution of the range-1 signal in Figure 10b. (**a**) The distribution for frequency f∈ [0, 1 Hz]. (**b**) The 1D representation of (**a**): The strongest peak of each time point. The mode value of the curve is marked in red.

If the test person slightly moved his body during the measurement, and the movement is greater than the chest movement caused by breathing, the FFT spectrum may be too noisy to give the correct respiration frequency. Time–frequency analysis can solve this problem, such as the short-time Fourier transform (STFT), which divides the time domain signal into short segments and then calculates the FFT. Among different time–frequency analysis methods, the continuous wavelet transform (CWT) method uses various time window sizes for different frequencies, thus providing high-frequency resolution for low-frequency signal components and high time resolution for high-frequency signal components. The discrete-continuous wavelet transform is

(19) $$X\left[u,s\right]=\sum_{n=0}^{N-1}x\left[n\right]\frac{1}{\sqrt{s}}\psi^{*}\left(\frac{n-u}{s}\right),$$

where ψ is the wavelet kernel function, *u* is the time shift variable, and *s* is the magnitude scaling variable. We use a generalized Morse wavelet ψ [43]. The generalized Morse wavelet is a family of wavelets that are exactly analytic and are defined in the frequency domain as [44]

(20) $$\Psi_{\beta ,\gamma }\left(\omega \right)=U\left(\omega \right)a_{\beta ,\gamma }\omega^{\beta }e^{-\omega^{\gamma }},$$

where U(ω) is the unit step function, $a_{\beta ,\gamma }$ is a normalization constant, and β and γ are two parameters that control the form of the Morse wavelet [44,45]. Figure 12a and Figure 13a are the time-frequency distribution calculated with CWT for the filtered range-2 and range-1 signals shown in Figure 10b, respectively.

The bright strip in Figure 12a indicates the person’s respiratory rate, which is relatively constant throughout the measurement. However, in Figure 13a, there is no continuous strong signal but discrete noise. To determine the respiration rate with CWT, we first define a measure called peak factor PF, which describes the size of a peak at a time point. Three coefficients describe the size of a peak: amplitude *A*, prominence *P*, and width *W* at P/2. They are all important, so we multiply them together and define the peak factor PF at time point *n* as PF[n]=A[n]·P[n]·W[n].

The frequency of the peak with the largest PF at time point *n* is $f(PF_{\max}\left[n\right])$. The $f(PF_{\max}\left[n\right])$ at each time point in the CWT distribution builds a 1D curve, as illustrated in Figure 12b and Figure 13b. We denote the most often occurring frequency value of the 1D curve, namely the mode, as the respiratory frequency estimated by CWT

(21) $$f_{\mathrm{resp},\mathrm{cwt}}=\mathrm{mode}\left(f\left(PF_{\max}\left[n\right]\right)\right).$$

We have used FFT and CWT, respectively, to estimate respiratory frequency. If these two results are very close, they mutually verify that they have detected the same signal. Now, we define the normalized difference between $f_{\mathrm{resp},\mathrm{cwt}}$ and $f_{\mathrm{resp},\mathrm{fft}}$ as

(22) $$\Delta f_{\mathrm{resp}}=\frac{|f_{\mathrm{resp},\mathrm{fft}}-f_{\mathrm{resp},\mathrm{cwt}}|}{(f_{\mathrm{res},\mathrm{fft}}+f_{\mathrm{res},\mathrm{cwt}})/2}\;\cdot \;100\%.$$

The signal prominence ratio $PR_{\mathrm{fft}}$ and $\Delta f_{\mathrm{resp}}$ are two metrics that we defined to describe the quality of a bioradar measurement. In Section 6, we will present some field measurements and evaluate them using these two measures.

## 5. Experiment with Two Persons

**Figure 14:**
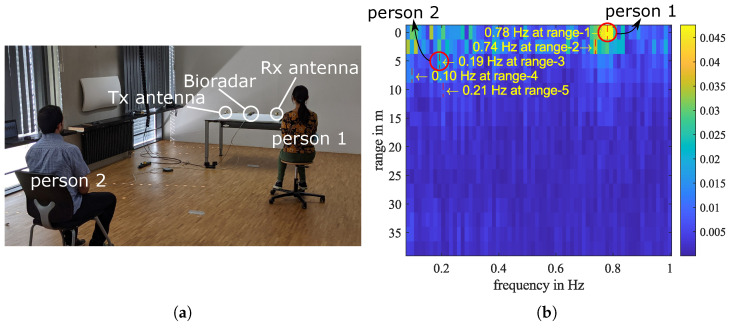
A bioradar experiment with two people. (**a**) Photograph of the experiment setup. Person 1 sits in the first range interval, and person 2 sits in the third range interval. (**b**) The breathing frequency–distance plot.

The system can detect multiple persons if they locate in different range intervals and breathe with different frequencies. An experiment with two persons is shown in Figure 14a. The first person sits in the first range interval, and the second person sits in the third range interval. The first person was instructed to breathe quickly. From the breathing frequency-distance plot, Figure 14b, we can see that person 1 has a breathing rate of about 0.78 Hz, while person 2 breathes normally with a rate of about 0.2 Hz. The multipath reflection of the second person is detected in range-5 with a weaker intensity.

## 6. Field Measurement

**Figure 15:**
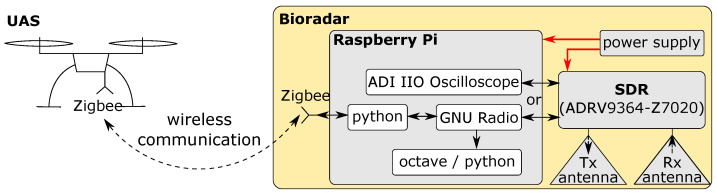
Block diagram of the prototype bioradar system. Relevant software used on the Raspberry Pi is illustrated with white blocks.

A standalone prototype bioradar system was built for the first field measurements. Figure 15 shows the system block diagram. A Raspberry Pi serves as the host computer of the SDR. The UAS communicates with the Raspberry Pi wirelessly via Zigbee. In the testing phase, we use ADI IIO Oscilloscope to configure the settings and visualize the real-time received signals in the time and frequency domain. For field measurements, we use GNU Radio to control the SDR. The GNU Radio code is started by python code when the Zigbee on the Pi receives a ’start’ command. The flowchart of the GNU Radio code can be found in Appendix A.

**Figure 16:**
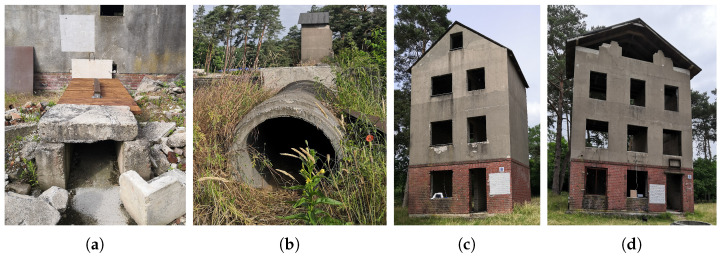
Measurement scenarios: (**a**) A tunnel covered with a wooden plate. (**b**) A concrete tube. (**c**) A building with wooden floors. The test person lay on the ground floor. The bioradar was placed on the first floor. (**d**) A building with reinforced concrete floors. The test person lay on the ground floor. The bioradar was placed on the first floor.

A series of field tests have been conducted in the training center for the German Federal Agency for Technical Relief (THW) in Wesel, Germany. As shown in Figure 16a–d, we selected four measurement scenarios with different building materials for bioradar measurements. It is safe to hide a test person in all the scenarios. In each case, two measurements were taken with one person hidden.

**Figure A2:**
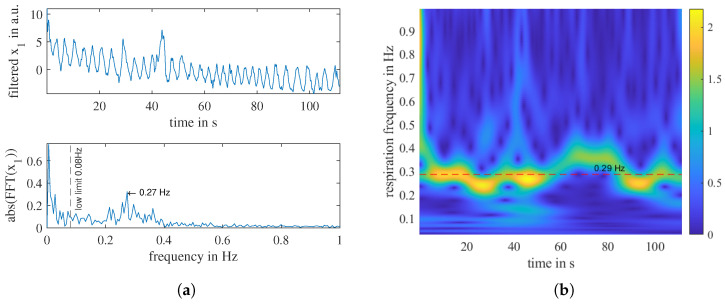
Measurement Nr.1 in scenario shown in Figure 16a: (**a**) top: preprocessed time-domain signal of the first range bin; bottom: FFT of the signal in the upper plot. (**b**) CWT of the signal in (**a**).

**Figure A3:**
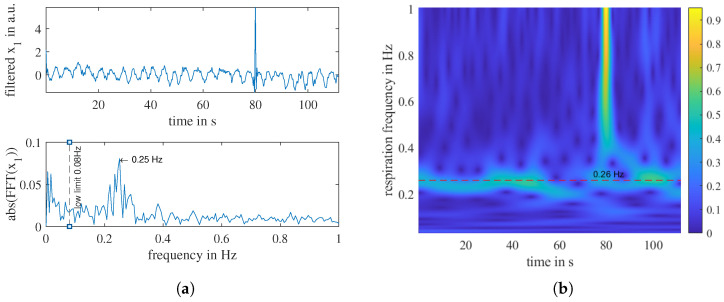
Measurement Nr.3 in scenario shown in Figure 16b: (**a**) top: preprocessed time-domain signal of the first range bin; bottom: FFT of the signal in the upper plot. (**b**) CWT of the signal in (**a**).

**Figure A4:**
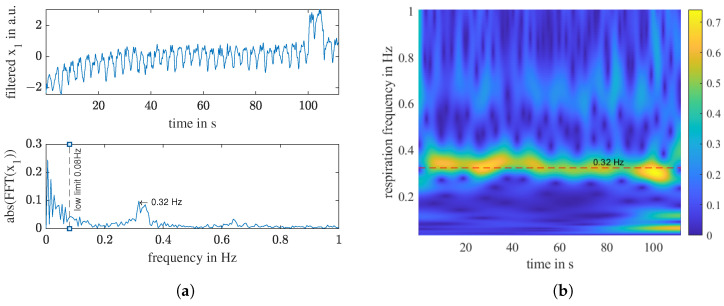
Measurement Nr.5 in scenario shown in Figure 16c: (**a**) top: preprocessed time-domain signal of the first range bin; bottom: FFT of the signal in the upper plot. (**b**) CWT of the signal in (**a**).

**Figure A5:**
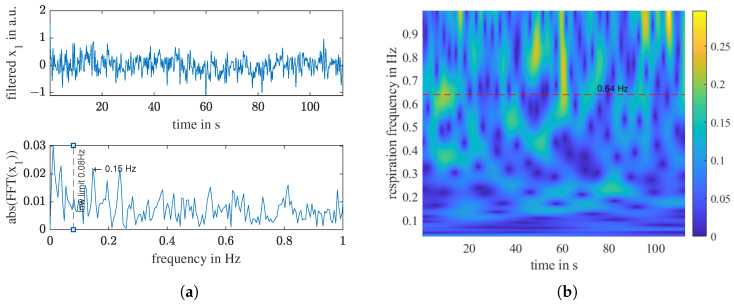
Measurement Nr.7 in scenario shown in Figure 16d: (**a**) top: preprocessed time-domain signal of the first range bin; bottom: FFT of the signal in the upper plot. (**b**) CWT of the signal in (**a**).

Here we only investigate the sub-signals of the first range interval. Figure A2, Figure A3, Figure A4 and Figure A5 in Appendix B present plots of one measurement in each case. The numerical results of the measurements are summarized in Table 3. The last column, whether life is detected, is based on observing the measurement results, assuming we do not know the presence of a person.

The bioradar can detect breathing well through wood, as shown in Figure A2 and Figure A4. Besides, the four measurements in scenes (a) and (c) show both high $PR_{\mathrm{fft}}$ and low $\Delta f_{\mathrm{resp}}$. Electromagnetic waves can also penetrate the concrete. However, the concrete pipe in (b) has a curved surface and can result in an air gap between the antenna and the concrete surface, and consequently, a poor $PR_{\mathrm{fft}}$, as in measurement 4. The detection is unstable, and breathing can be observed in measurement 3 but not in measurement 4. The most difficult case is the scene (d) because reinforced concrete has a metal mesh that shields electromagnetic waves. As expected, the measurements in scene (d) exhibit just noise, as shown in Figure A5. Both measurements 7 and 8 have a low $PR_{\mathrm{fft}}$ and a large $\Delta f_{\mathrm{resp}}$.

For these eight measurements, $\Delta f_{\mathrm{resp}}$ is as good a detection metric as $PR_{\mathrm{fft}}$. In all measurements in which life was detected, $PR_{\mathrm{fft}}>$ 17.4 dB and $\Delta f_{\mathrm{resp}}<7.2\%$. In all measurements that failed to detect life, $PR_{\mathrm{fft}}<$ 13 dB and $\Delta f_{\mathrm{resp}}>28.5\%$. Therefore, for future measurements, we can use $PR_{\mathrm{fft}}=$ 15 dB and $\Delta f_{\mathrm{resp}}=10\%$ as thresholds to make the decision.

## 7. Conclusions

A ground-penetrating bioradar using frequency comb continuous wave is presented. The bioradar system is implemented based on the commercial SDR ADRV9364-Z7020 and operates in the 1.3 GHz band. The frequency comb is software-defined, and the parameters can be changed easily for different target detection applications. We have introduced the signal processing of the returned frequency comb signal in detail. The developed bioradar can detect the respiration of an alive person through different nonmetallic building materials. Two metrics are defined to evaluate the detection quality. We conducted eight ground-penetrating field measurements with the prototype system.

In the proposed method, the LO as a key component decides the accuracy of the transmitted and received combs. Therefore, an LO with reduced phase noise, such as an opto-electronic oscillator applied in a synthetic aperture radar [46], could be used in future iterations of the system to reduce the noise figure in the comb and improve the detection. Moreover, to compare different radar schemes, like FMCW or SFCW, with the proposed FCCW radar, the signal-to-noise ratios of the respective methods should be compared. Because it is difficult to compare them in exactly the same hardware, we plan to investigate this with more detailed simulation studies.

In future work, more measurements with different test persons and control measurements without persons should be performed to validate the system’s capabilities. Moreover, we are currently investigating the returned signal to extract more metrics, i.e., features in machine-learning terminology. With more measurements, these features can train a machine-learning model, such as a support vector machine, to classify breathing detection automatically. 

## Figures and Tables

**Table 1:** Specifications used for the frequency comb.

Parameter	Symbol	Value
sampling frequency	$f_{s}$	61.44 MHz
number of tones in the frequency comb	*M*	32
number of samples in waveform s[l]	*L*	4096
bandwidth	BW	59.52 MHz
start frequency	$f_{\mathrm{tx}}\left[1\right]$	−29.28 MHz
stop frequency	$f_{\mathrm{tx}}\left[M\right]$	30.24 MHz

**Table 2:** System parameters.

Parameter	Symbol	Value
sampling frequency	$f_{s}$	61.44 MHz
LO-frequency of the receiver	$f_{\mathrm{Rx}}$	1.3 GHz
LO-frequency of the transmitter	$f_{\mathrm{Tx}}$	1.3007 GHz
transmitting waveform	s[l]	see Equation (5) and Table 1
number of samples per receiving frame	*L*	4096
number of frames	*N*	20k
frame rate	$f_{s,frame}$	225 Hz
measurement duration	$T_{\mathrm{meas}}$	about 86 s

**Table 3:** Evaluation of the measurement results.

Nr.	Scenario	$f_{\mathrm{resp},\mathrm{fft}}$	$f_{\mathrm{resp},\mathrm{cwt}}$	${\mathrm{PR}}_{\mathrm{fft}}$	$\Delta f_{\mathrm{resp}}$	Life Detected
1	(a)	0.27 Hz	0.29 Hz	17.45 dB	7.1%	yes
2	(a)	0.30 Hz	0.30 Hz	17.94 dB	0%	yes
3	(b)	0.25 Hz	0.26 Hz	18.25 dB	0.4%	yes
4	(b)	0.13 Hz	0.27 Hz	7.35 dB	70%	no
5	(c)	0.32 Hz	0.32 Hz	22.35 dB	0%	yes
6	(c)	0.13 Hz	0.13 Hz	21.02 dB	0%	yes
7	(d)	0.15 Hz	0.64 Hz	12.52 dB	124%	no
8	(d)	0.09 Hz	0.12 Hz	12.99 dB	28.6%	no

**Figure A1:**
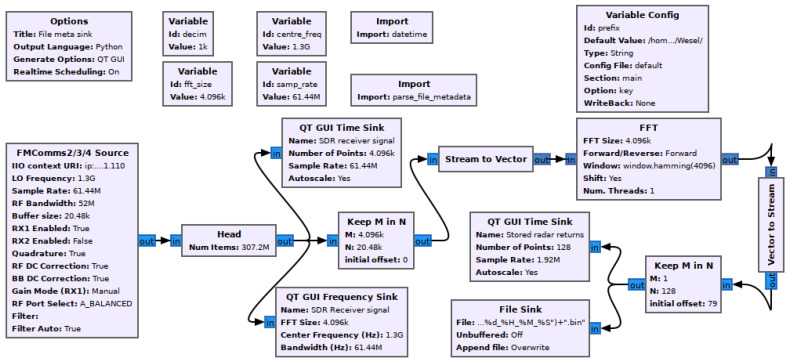
GNU radio flow graph.

## Data Availability

Data is contained within the article.
